# Nomophobia in university students during COVID-19 outbreak: a cross-sectional study

**DOI:** 10.3389/fpubh.2023.1242092

**Published:** 2023-09-22

**Authors:** Noelia Navas-Echazarreta, Raúl Juárez-Vela, Ana Belén Subirón-Valera, Beatriz Rodríguez-Roca, Isabel Antón-Solanas, María Teresa Fernández-Rodrigo, Regina Ruiz de Viñaspre-Hernandez, Antonio Martínez Sabater, Emmanuel Echániz-Serrano, Carles Saus-Ortega, Iván Santolalla-Arnedo, Pedro José Satústegui-Dordá

**Affiliations:** ^1^Doctoral Program in Health Sciences and Sports, University of Zaragoza, Zaragoza, Aragon, Spain; ^2^Faculty of Health Sciences, University of La Rioja, Logroño, La Rioja, Spain; ^3^Grupac Research Group, Department of Nursing, University of La Rioja, Logroño, La Rioja, Spain; ^4^Faculty of Health Sciences, University of La Rioja, Logroño, Spain; ^5^SAPIENF (B53_23R) Research Group, Faculty of Health Sciences, University of Zaragoza, Zaragoza, Spain; ^6^Faculty of Nursing, University of Valencia, Valencia, Spain; ^7^University of Valencia, Adscript Centre of la Fe, Research Group in Art and Science in Care, Institute for Health Research La Fe (IISLAFE), University School of Nursing La Fe, Valencia, Spain

**Keywords:** smartphones, nomophobia, students, gender, dependence

## Abstract

**Introduction:**

Currently, access to the Internet through smartphones has led to their functions going beyond purely communicative ones, allowing the management of massive, instantaneous, and easily accessible information. This research analyzed the differences in smartphone use and the prevalence of nomophobia, mainly according to gender and university degree of Health Sciences students at the University of Zaragoza during the COVID-19 confinement in Spain.

**Methods:**

A descriptive cross-sectional study was carried out on a sample of 318 first and second-grade students, who completed an online questionnaire sent to their institutional email, which included sociodemographic questions, other questions about smartphone use, and the Nomophobia Questionnaire (NMP-Q) scale.

**Results:**

Compared to men (*n* = 58), women (*n* = 260) were more likely to use their smartphones more intensively daily, as were occupational therapy students compared to the other degree programs studied. The prevalence of nomophobia was moderate, being around the risk of suffering from it. No significant differences in scores for nomophobia among students were found according to gender, university degree, or population nucleus for the nomophobia scores of the students.

**Discussion:**

The present study extends the existing literature on nomophobia by providing results of interest in terms of gender and the exceptional healthcare context of COVID-19. The results suggest that despite intense daily smartphone use young people did not reach severe nomophobia figures. This fact underlines the need for appropriate and healthy technology education. Understanding the characteristics of the populations that use the smartphone the most may help to analyze nomophobia rates and the massive use of the device.

## 1. Introduction

Currently, access to the Internet through smartphones has led to their functions going beyond purely communicative ones, allowing the management of massive, instantaneous, and easily accessible information. More and more young people are acquiring a smartphone at an early age (1–4 years). Home confinement by COVID-19 imposed the need to adapt to the new reality using technology to ensure that private and working life could continue under health restrictions. Smartphones, more than ever, have been an excellent tool for communication and information management, which has boosted the use of these devices in all areas of daily life (1, 2).

Smartphones give individuals responsibility for their health, education, security, home, etc., assuming the position of basic goods that can improve daily routines by downloading Apps and, at the same time, help to cope with the emotional burden of the pandemic (2, 3). Thus, they promote better use of resources by housing multiple functions in a single device (4). However, the advantages offered by using smartphones can lead to the need for and pathological addiction to this device, which can lead to a pathology known as nomophobia.

Nomophobia disorder manifests as an irrational fear or feeling of anxiety at not having one’s phone, not using it, running out of battery, or not having or losing an internet connection, which deprives individuals of constant communication (2, 5). Although nomophobia is not marked in the Diagnostic and Statistical Manual of Mental Disorders (DSM-V), it is commonly perceived with the phobia criteria outlined in the DSM-V such as excessive and unreasonable fear or anxiety associated with an unintended object or situation, which causes immediate anxiety. Despite the person recognizing that her fear is disproportionate and avoidance of the feared situations, at the same time, we can observe disruption of routines and relationships due to the phobia. In this case, in 2014, Bragazzi and Del Puente, proposed to consider the inclusion of nomophobia in the DSM-V given its clinical relevance, epidemiological characteristics, and the importance of this condition shortly, as evidenced (6).

The possibility offered by smartphones to obtain easily accessible communication over time encourages people to be permanently connected and can lead to an addiction problem Young people use smartphones much more intensively than other sectors of the population, as social and interpersonal relationship needs take on central importance. This population group is therefore much more prone and vulnerable to smartphone-related problems (7–9). In addition, social networks aggravate problematic use. According to the latest research (2, 10–12), there are differences between men and women in the time spent using smartphones, which is predominantly intense and constant. In addition, the exceptional situation brought about by the arrival of COVID-19 led to new forms of smartphone use by young university students who were in a situation of home confinement (2).

Age and gender have also been indicated in numerous studies as conditioning factors in nomophobia. Likewise, young women score higher on the scales for this disorder, due in part to their greater use of social media platforms. However, smartphone use is becoming increasingly homogeneous across individuals, which casts aside the classic gender roles set by the collective imagination (13, 14).

Regarding this last point, it is worth noting that the scientific literature and studies on technology use by young people have been lacking over the last decade in comparison to the burgeoning current research (15–17). There is currently much controversy regarding gender as a modulating factor in nomophobia. There are previous studies that show differences between men and women in nomophobia, with women being more likely to have this disorder (12, 18, 19). Similarly, there are studies in which no differences were found according to gender (14, 20, 21). In this sense, it is interesting to know whether Health Sciences students suffer from nomophobia and whether there are differences based on gender.

The study aims to describe the use of Mobil devices and to analyze the presence of nomophobia in first and second-year university students in the Faculty of Health Sciences (FHS) at the University of Zaragoza, with stratification of the results by gender, degree, and population nucleus where they usually live.

## 2. Materials and methods

### 2.1. Study design

We performed an observational, descriptive, cross-sectional study during April 2020. The study complies with the STROBE checklist for cross-sectional studies.

### 2.2. Population and scope of the study

The study was conducted at the FHS of the University of Zaragoza (Spain) with university students enrolled in their first and second year at the faculty who fully and correctly answered the questionnaire. No probabilistic purposive sampling by the investigators was used. The results were obtained during the questionnaire carried out in April 2020. Of the total number of students that made up the study population (*N* = 610), the sample calculation for a confidence level of 95% and a margin of error of 5% was 237 students. The final sample consisted of 318 participants.

Inclusion criteria refer to the student in the faculty of health sciences of the University of Zaragoza, with informed consent signed, and exclusion criteria were those participants with incomplete or incorrectly completed questionnaires which, therefore, were not useful for the study.

### 2.3. Data collection instrument or procedure

Due to the lockdown ordered by the Government of Spain as a consequence COVID-19 outbreak, information was gathered using an online form on the Google Apps For Education (GAFE) platform. It is a package of Google tools and services designed for educational centers and allows collaboration and learning safely and securely. In the present investigation, this tool was accepted by the Research Ethics Committee of the Community of Aragón and the University of Zaragoza, Spain. The data were protected and each student could only access the questionnaire once. Participants received an email containing the survey to their university email account, which remained freely open for participants throughout April 2020. The email contained a link that directed them to the questionnaire on the GAFE platform. When they accessed the questionnaire, participants were asked to sign their consent to participate in the study and were given a brief explanation of the guidelines to continue and access it. The questionnaires were registered and coded with random numbers and letters.

Nomophobia was assessed using the Spanish version of the Nomophobia Questionnaire (NMP-Q) scale (22). With an ordinal alpha of 0.95 for the total score, this scale has 20 items, with 7 answer options (from 1 = strongly disagree to 7 = strongly agree). Scores range from 20 to 140, with which higher scores are linked to higher levels of nomophobia. The 15th percentile pertains to occasional users or not nomophobia (with a cut-off score of 34 points), the risk zone is from the 80th percentile (with a cut-off score of 72 points), whilst problematic smartphone use starts at the 95th percentile (94 points). The Nomophobia Questionnaire (NMP-Q) in its Spanish version (22) confirms the factorial structure of the Spanish version of the NMP-Q of Gonzalez-Cabrera et al. (23) and the original four-factor model of Yildirimy and Correia (24) conducted through a non-probabilistic sampling on 5,012 participants, showing a hierarchical model of 4 factors with good psychometric fit.

### 2.4. Study variables

Primary variables included questions on student sociodemographic characteristics (gender, age, family home postcode), academic education (year and degree), and their smartphone (operating system, time spent using it daily, daily dependence, and overall smartphone dependence). The second variable includes questions about NMP.

### 2.5. Statistical procedures

Descriptive statistics (mean, standard deviation, median, coefficient of variation, and asymmetry were used to report quantitative values). The distribution of categorical variables is shown by absolute and relative frequencies.

Distribution normality was verified using the Shapiro–Wilk test. The accepted significance value throughout the entire study was 0.05, assuming a confidence interval of 95%. Accordingly, values below 0.05 (*p* < 0.05) were considered significant. A univariate and bivariate analysis was performed. In the bivariate analysis, the relationship between the quantitative and qualitative variables was studied. The mean difference of the continuous variables (age and nomophobia) was compared. In the cases where at least one quantitative variable was compared, after rejecting the normality of the groups, a nonparametric equivalence Mann–Whitney U test was conducted, which yielded the value of p for the compared groups. Where the surveyed population was stratified into three groups, first the normality and equality of variances were tested. Next, an ANOVA test was run for equal variances, and a Kruskal–Wallis test was to determine if there were variations from the normality. The analysis was performed using the RCommander program (version 3.6.1).

### 2.6. Ethical considerations

This study was developed following the Data Protection Regulation (EU) 2016/679 of the European Parliament and the Organic Law 3/2018 on data protection. The information was treated confidentially and anonymously as the data were dissociated. The study was approved by the ethics committee of Aragon (CEICA-PI 20/170).

## 3. Results

### 3.1. Sociodemographic characteristics

The sociodemographic characteristics of the population under study are shown in Table 1. They indicated that 81.8% of the surveyed students were women and 18.2% were men. 79.56% of the students were from a city, whilst 20.44% had their normal residence in a village. The mean age was 20.4 years (men = 20.2; women = 20.5).

**Table 1:** Descriptive analysis of the sociodemographic variables: population nucleus and university degree according to gender.

Variables		Women		Men		No binary		Total	
Type	Subtype	*N*	%	*N*	%	*N*	%	*N*	%
Total	Gender	260	81.80	58	18.20	0	0	318	100.00
Population nucleus									
City		206	81.42	46	18.18	0	0	252	79.56
Village		54	83.07	12	18.46	0	0	66	20.44
Degree									
Nursing		118	87.40	17	12.60	0	0	135	42.50
Occupational Therapy		73	85.90	12	14.10	0	0	85	26.70
Physiotherapy		69	70.40	29	29.60	0	0	98	30.80

N, population.

There was a clear predominance of female students at a general level, which was maintained in all the degrees based in the Faculty of Health Sciences. However, there were notable differences between the physiotherapy degree, with a masculinity ratio of 0.4, and the occupational therapy or nursing degrees where the masculinity ratios were between 0.16 and 0.14, respectively.

### 3.2. Smartphone use and risk of nomophobia

The students used their smartphones for approximately 4 h per day (4.22 h) and checked them on average every 35 min (0.58 h) as shown in Table 2. The analyses (Table 3) showed that men spent a little over 3 and a half hours per day on their smartphone, whilst women did so for a longer average period of 4 h and 35 min (Wilcoxon signed-rank test, *p* = 0.001) as well as Occupational Therapy students (ANOVA, *p* = 0.012) who reported using their smartphone an average of 4.75 h per day.

**Table 2:** Daily smartphone usage time and smartphone checking-in hours.

Variables	*M*	Mdn	SD	CV	S	*N*
Smartphone usage time	4.22	4	1.95	0.46	1.28	318
Smartphone checking time	0.58	0.38	0.66	1.11	2.76	318

M, mean; Mdn, Median; SD, Standard Deviation; CV, coefficient of variation; S, Skewness.

**Table 3:** Daily smartphone usage time according to gender, university degree, and population nucleus.

Variables		*M*	Mdn	SD	CV	S	*N*	Test
Gender								
	Men	3.65	3	1.92	0.53	1.63	58	Wilcoxon *p* = 0.001
	Women	4.35	4	1.94	0.45	1.27	260	
Degree								
	Nursing	4.09	4	1.59	0.39	0.84	135	ANOVA *p* = 0.012
	Occupational therapy	4.75	4	2.06	0.43	1.03	85	
	Physiotherapy	3.95	3,5	2.22	0.56	1.71	98	
Population nucleus								
	Village	4.35	4	2.03	0.47	1.65	65	Wilcoxon *p* = 0.810
	City	4.18	4	1.93	0.46	1.81	253	

M, mean; Mdn, Median; SD, Standard Deviation; CV, coefficient of variation; S, Skewness.

On the other hand, 86.48% of students stated they were able to spend 24 h without their smartphone, although almost the same percentage (84.2%) stated they would not be able to go without it indefinitely. Nevertheless, a higher percentage of female students (15.4%) than male students (5.2%) reported daily dependence on their smartphones (chi-squared test, *p* = 0.039). On the other hand, they were not found by a degree or population nucleus (Table 4).

**Table 4:** Students perceived diary dependence on their smartphones.

Variables	No	Yes	Test
Gender			
Men	94.8%	5.2%	$x^{2}$ *p* = 0.039
Women	84.6%	15.4%	
Degree			
Nursing	81.5%	18.5%	$x^{2}$ *p* = 0.0686
Physiotherapy	91.8%	8.2%	
Occupational therapy	88.8%	11.2%	
Population nucleus			
Village	83.1%	16.9%	$x^{2}$ *p* = 0.369
City	87.4%	12.6%	

**Figure 1:**
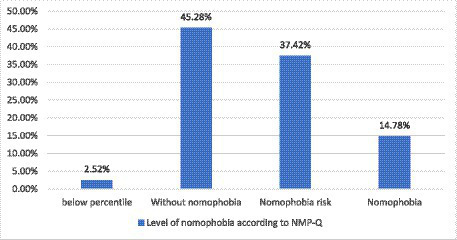
Classification of nomophobia levels according to the Nomophobia Questionnaire (NMP-Q) Spanish version.

According to the rating of NMP-Q, it was observed that rating between 34–72 there is no nomophobia, 73–94 risk of nomophobia, and 100–140 nomophobia. After analyzing the student scores on the NMP-Q scale, it was observed that 45.28% of the population in the study were not homophobic, 37.42% were in the risk zone and 14.78% of the students had nomophobia (Figure 1).

### 3.3. Relationship between gender, degree, and population nucleus

Table 5 shows the results of the analysis of the nomophobia variable in the NMP-Q scale. The results are broken down by student gender, university degree, and population nucleus where they usually live. Regarding gender, the mean score was higher in women than in their male peers, with a maximum score of 132 points compared to a maximum of 119 points in men. However, no significant differences were identified in nomophobia scores based on student sex (Welch’s t-test, *p* = 0.602).

**Table 5:** Mean nomophobia score (NMP-Q) according to gender, university degree, and population nucleus.

Variables	*M*	Mdn	SD	CV	S	min	max	N	Test
General	71.7	72	21.3	0.30	0,13	20	132	318	–
Gender									
Men	70.5	66.5	19.0	0.27	0.33	36	119	58	Welch *p* = 0.602
Women	72.0	73	21.8	0.30	0.08	20	132	260	
Degree									
Nursing	73.6	73	20.4	0.28	0.15	28	124	135	ANOVA *p* = 0.618
Physiotherapy	70.4	74	22.4	0.32	−0.2	20	113	85	
Occupational therapy	70.2	71	21.5	0.31	0.48	35	132	98	
Population nucleus									
Village	72.34	73	22.70	0.31	5e^−3^	23	132	65	Welch *p* = 0.8071
City	71.5	72	20.9	0.29	0.16	20	129	253	

M, mean; Mdn, Median; SD, Standard Deviation; CV, coefficient of variation; S, Skewness.

Related to the university degrees that were present in the study were degrees in nursing (*n* = 135), physiotherapy (*n* = 85), and occupational therapy (*n* = 80). Nursing students presented a mean score of 73.66 points in the NMP-Q, future occupational therapists had 70.42 points, and physiotherapists had 70.21 points. The data shows that no significant differences were found for nomophobia scores according to the degree (ANOVA, *p* = 0.618). According to the type of population nucleus in which the students usually resided, the results showed no significant differences in their score on the NMP-Q scale (Welch’s *t*-test, *p* = 0.807).

## 4. Discussion

The COVID-19 confinement provided an opportunity to investigate the prevalence of nomophobia among health science students during a period of health crisis.

The smartphone is currently the device of choice, as it allows information to be obtained and communicated continuously and instantaneously. This communicative function contributes to the satisfaction of individuals’ need for social relationships (25, 26). Especially during late adolescence (17–21 years) and later youth, interpersonal relationships acquire greater relevance, as they favor the feeling of acceptance by the social group (27). Young people use more intensive the smartphone, which is reflected in the hours of use of the device and the constant checking of it (28).

Along the same lines as the present investigation, several studies showed high results regarding the time spent using smartphones by young people (28, 29). The COVID-19 pandemic highlighted the need to use technology to maintain communication with the rest of society. University students manage their daily activities autonomously and rely mainly on their smartphones for this purpose (30, 31).

As in the present study, the time that young people use their smartphones daily exceeds 3 h in most studies (32–35) and even exceeds 5 h (36). Most studies agree that women tend to use their smartphones more daily for socializing (37–39). In addition, there is an underestimation of smartphone usage time among students, since, as indicated by Cabrera García-Ochoa (34) university students claim to use the smartphone between 2 and 4 h per day and, however, they claim to feel annoyed for not being able to check the device in class. Checking the smartphone is done routinely and automatically, creating the false perception of less exposure time to the device (34, 40).

In this way, the constant use of the device and its main function in the lives of young people can lead to its dependence and the development of affectations such as nomophobia (2, 30).

Analysis of the prevalence of nomophobia during the month which coincided with the strictest home confinement measures in Spain, provides insight into the prevalence of this disorder at a time when information and communication technologies were a key element in being able to maintain educational, work, and personal activity (31).

Coinciding with the above results, the works of Daei et al. (13), Cain et al. (41), and Moreno Guerrero et al. (13, 42) showed moderate nomophobia rates very similar to those of the present investigation. In some studies, this proportion tended to increase (13). The elevated risk of nomophobia among young people prevails over milder forms of dependence.

It has been observed that the nomophobia score increases especially when there are situations that make interpersonal relationships impossible, such as the impossibility of contacting or being contacted by family and friends (42). The need for social relationships that the smartphone allows satisfying through its various functionalities is the factor that drives young people to make intensive use of this technology to expand their relational sphere (9). According to Braña Sánchez and Moral Jiménez (43), greater use of the phone can predict an increased risk of nomophobia, as well as generalized anxiety. Mainly, the authors relate these factors to dependence on social networks, which leads individuals to need their smartphones excessively, in turn generating disorders such as nomophobia.

As in several studies (41, 42, 44), even though the highest nomophobia scores on the Nomophobia Questionnaire (NMP-Q) scale were recorded by women, no significant differences were found with their male peers. The results of the present investigation contribute to the existing literature on nomophobia, which presents discrepancies in terms of gender (12, 14, 18, 20, 21). In Spain, the majority of university Health Sciences studies are attended by women, with rates of around 80%. This fact implies that in the present research, a population sample with homogeneous characteristics was obtained (45).

Regarding the different university degrees studied, the future nurses obtained a mean score of nomophobia that exceeded the risk percentile on the NMP-Q scale without any significant differences with the Occupational Therapy and Physiotherapy students. Similarly, nomophobia in Health Sciences studies is high, especially in the Nursing degree (46). Berdida and Grande (47), in their research with nursing students, found a relationship between their nomophobia and a lower level of attention and motivation. Nomophobia represented a danger to their health, but also to their patients. One of the main functions of future health professionals is vigilance and clinical safety, which was altered when attention was reduced by the urgent need to consult the smartphone (47).

The COVID-19 pandemic made it impossible for students acquisition of practical skills, to attend physical classes and contributed to the transition to the implementation of all their educational activities through distance learning technologies (30). During this health crisis, in which information on the evolution of COVID-19 was mainly obtained through the smartphone, reinforces the results obtained in the present investigation of the extensive time spent using the smartphone. COVID-19 signified a risk of suffering from nomophobia in students. The results obtained showed a moderate nomophobia score as in similar studies on the university population (13, 41). Reduced face-to-face social interactions generated a context of high anxiety and intense smartphone use that contributed to the development of nomophobia during this period (48).

## 5. Strengths and limitations

One of the limitations was that almost 82% of the participants were women in a study stratified by gender, which is an enormous limitation. Although multiple comparisons analysis provides confidence in the results of the present study, future research should include a more heterogeneous sample so that comparisons by gender have more statistical power. In addition, although there were no age differences among the participants, it would be interesting to conduct future studies that include larger age ranges. However, due to the exploratory nature of this study, these results are considered a preliminary test for future research to address the problem of nomophobia among the young university population. Furthermore, it offers a reading of a current social disorder at a time of epidemiological crisis. Specifically, future work comparing the results before and after the COVID-19 health crisis in similar populations would be beneficial.

## 6. Conclusion

The study indicated an intensive use of the smartphone that can be classified as dysfunctional, with a large proportion of health science students showing a risk of suffering nomophobia. On the other hand, it should be noted that the students of the different degrees in the research did not use their smartphones in the same way, with daily use being higher in occupational therapy students. In any case, concerning the rates of nomophobia presented by the students, no significant differences were found by gender. Likewise, no relationship was found between nomophobia and the degree and the type of population center to which the students’ normal residence belonged.

Increasingly, relationships between individuals and between individuals and institutions are mediated by electronic devices. In this context, it is necessary to know whether this relationship with technology is healthy or could turn into a health problem. For this reason, the research focuses on future health professionals, who will be in charge of following the trajectory of nomophobia and establishing measures or action plans according to the current situation. To this end, future research related to this disorder is required, including a more heterogeneous population with a larger sample size.

## 7. Implication for practice

This fact reveals a unifying break and identifies smartphone use, as popular as it is necessary for young people, as a common, homogenous trait. These findings may contribute to a better understanding of the use of mobile phones, especially when the time spent on and commitment to them may increase. Intense and widespread use of smartphones by young people contributes to the need to improve the technological education of this population group, orienting the guidelines to the healthy use of smartphones. Prevention of nomophobia and health promotion are key for educational projects to contribute to reinforcing healthy technological habits in the present social reality in which young people live in cyberspace.

## Data availability statement

The raw data supporting the conclusions of this article will be made available by the authors, without undue reservation.

## Ethics statement

The studies involving humans were approved by The ethics committee of Aragon (CEICA-PI 20/170). The studies were conducted in accordance with the local legislation and institutional requirements. The participants provided their written informed consent to participate in this study.

## Author contributions

NN-E: conceptualization. RJ-V: methodology. AS-V and BR-R: software and validation. IA-S, MF-R, and RV-H: formal analysis. AS, EE-S, and CS-O: investigation. IS-A: resources. PS-D: data curation. NN-E, IA-S, MF-R, and RV-H: writing—original draft preparation. AS-V, BR-R, AS, EE-S, and CS-O: writing—review and editing. RJ-V and PS-D: supervision. All authors contributed to the article and approved the submitted version.

## Conflict of interest

The authors declare that the research was conducted in the absence of any commercial or financial relationships that could be construed as a potential conflict of interest.

## Publisher’s note

All claims expressed in this article are solely those of the authors and do not necessarily represent those of their affiliated organizations, or those of the publisher, the editors and the reviewers. Any product that may be evaluated in this article, or claim that may be made by its manufacturer, is not guaranteed or endorsed by the publisher.
